# Outcome of Patients With Metastatic Lung Neuroendocrine Tumors Submitted to First Line Monotherapy With Somatostatin Analogs

**DOI:** 10.3389/fendo.2021.669484

**Published:** 2021-04-27

**Authors:** Elisa Lenotti, Andrea Alberti, Francesca Spada, Vito Amoroso, Patrick Maisonneuve, Salvatore Grisanti, Alice Baggi, Susanna Bianchi, Nicola Fazio, Alfredo Berruti

**Affiliations:** ^1^ Medical Oncology, Department of Medical and Surgical Specialties, Radiological Sciences and Public Health University of Brescia, ASST-Spedali Civili, Brescia, Italy; ^2^ Division of Gastrointestinal Medical Oncology and Neuroendocrine Tumors, IEO European Institute of Oncology, IRCCS, Milan, Italy; ^3^ Division of Epidemiology and Biostatistics, IEO European Institute of Oncology, IRCCS, Milan, Italy

**Keywords:** lung carcinoid, somatostatin analog, carcinoid syndrome, prognostic factor, distant metastases

## Abstract

**Objective:**

Antiproliferative activity of somatostatin analogs (SSAs) has been demonstrated in digestive neuroendocrine tumors (NETs), but few data have been published in patients with pulmonary NETs. We therefore conducted a retrospective study to provide additional data on the outcome of patients with metastatic lung NETs submitted to front line SSAs.

**Research Design and Methods:**

Patients with metastatic lung NET treated with first line SSA-monotherapy (octreotide or lanreotide) in two different reference Institutions were reviewed. Outcome measures were progression-free survival (PFS) overall survival (OS), overall response rate and safety. We also explored prognostic factors associated with PFS.

**Methods:**

The outcome of consecutive patients (pts) with metastatic lung NETs, who underwent first-line treatment with SSAs, recruited from 2014 on 2019 in two Italian reference Institutions, was retrospectively evaluated.

**Results:**

Thirty-one patients entered the study: 14 (45.2%) with typical and 17 (54.8%) atypical carcinoid. Six patients (19.4%) had a carcinoid syndrome. 60.0% of patients had Ki-67 ≤ 10%. Two (6.5%) patients obtained a partial response, 24 (77.4%) disease stabilization while 5 (16.1%) had progressive disease. Median progression free survival (PFS) was 28.6 months, median overall survival (OS) was not attained. Ki-67 ≤ 10%, typical carcinoid histotype and non-functioning disease, were associated with a non-significant PFS prolongation. PFS in patients with atypical carcinoids and in those with Ki-67 >10% was greater than 19 months.

**Conclusions:**

The long PFS and OS obtained in this case series suggest that SSAs could be effective as first line approach in the management of patients with progressive, metastatic pulmonary NET.

## Introduction

Bronchial carcinoids are a rare group of well-differentiated lung neuroendocrine tumors (NETs) with an incidence in western countries ranging from 0.2 to 2/100 000 persons/year (1). They are distinguished from neuroendocrine large and small cell lung carcinomas by histologic features as well as clinical, epidemiologic, genetic and prognostic parameters (2–8). According to WHO histologic criteria (9), lung NETs are divided in typical (low-grade, <2 mitoses/10 HPF and no necrosis) or atypical (intermediate grade, 2-10 mitosis/10HPF and/or foci of necrosis) carcinoids. Surgery is the mainstay of therapy for patients with disease at an early stage. Typical lung NETs have an excellent prognosis following surgical resection while atypical carcinoids are associated with a higher risk of disease relapse and a worse prognosis (10–12).

In patients with unresectable locally advanced or metastatic disease the goals of medical management are to control both hormone-related symptoms and tumor growth (13). Lung NETs frequently express somatostatin receptors, therefore somatostatin analogs (SSAs), such as lanreotide autogel (LAN) and octreotide long-acting release (LAR), are frequently used therapies and recommended by available guidelines (2, 13, 14). These drugs are well tolerated and have demonstrated both anti-secretory and anti-proliferative effects in NET patients. However, the data regarding the efficacy of SSAs in the management of lung carcinoids are very limited and refer to retrospective series (15–19). No prospective study data is currently available. The SPINET trial (NCT02683941), a placebo-controlled randomised phase III study, which was designed to evaluate lanreotide 120 mg in advanced lung carcinoids, was early stopped for insufficient enrolment (20). Most of the published retrospective studies are heterogeneous and included patients with NET of different primaries (21–28). Only 2 studies evaluated patients with lung carcinoids only. One of them enrolled patients undergoing SSAs both as first, and second line approach (21), only one study retrospectively evaluated patients treated with first line SSAs (22).

More data on the efficacy of SSAs in patients with advanced lung carcinoids, not previously submitted to systemic antineoplastic therapies, are therefore needed.

This retrospective study was designed to provide additional data on the efficacy of SSAs as first line therapy in patients with metastatic lung carcinoids.

## Material and Methods

We retrospectively reviewed data of consecutive patients with metastatic lung NET, treated with first line SSA monotherapy, from January 2014 to December 2019, at the Medical Oncology Unit of ASST Spedali Civili di Brescia and at the Division of Gastrointestinal Medical Oncology and Neuroendocrine Tumors of the European Institute of Oncology of Milan (IEO). To be included in the study the patients had to meet the following inclusion criteria: histopathological confirmed diagnosis of Lung NET according to 2015 WHO criteria (22), locally advanced or metastatic disease not amenable to radical surgery, absence of prior systemic therapy, at least three months of SSA therapy. Poorly differentiated morphology was an exclusion criterion. A single investigator (EL) collected clinical data from medical records. Database included the following data at baseline conditions: age, gender, tobacco exposure, surgery of primary tumor, histological subtype according 2015 WHO criteria, Ki- 67 index, mitotic count, hereditary or sporadic, primary tumor site, TNM/AJCC (tumor-node-metastasis/American Joint Committee on Cancer 2010 stage) (9), site of metastasis, functional tumor status. The following data were collected before and during the SSA therapy: ECOG PS, disease response at imaging according to RECIST criteria, type of SSA used and dose, date of initiation and completion of SSAs, adverse effects, any local therapies before SSA initiation or during SSA treatment. The study was approved by the institutional review board.

Follow-up visits during SSA therapy were performed approximately every 3 months and included a physical examination, complete blood count and biochemical profile. The evaluation of tumor response was assessed approximately every 6 months. Computed tomography [CT] scan or Positron emission tomography [PET] with Gallium 68 were the imaging techniques employed. The best overall response was defined according to the RECIST 1.1 criteria. Adverse events were evaluated over the whole duration of SSA administration and classified according to CTCAE v4.0.

Treatment with SSAs consisted in an intramuscular injection of octreotide long-acting release (LAR) (at dose of 20 mg or 30 mg every 4 weeks, or 30 mg every 3 weeks) or a subcutaneous injection of lanreotide depot (at dose of 60 mg or 120 mg every 4 weeks).

The primary study end point was progression free survival (PFS), defined as the time elapsing from SSA initiation to disease progression or death whichever occurred first. Patients with no event and alive were censored at the date of the last follow-up. Secondary endpoints were: overall survival (OS), defined as the time from SSA initiation to death from any cause. Survivors were censored at the date of their last follow-up; best overall response under SSA; toxicity.

### Statistical Analysis

**Figure 1:**
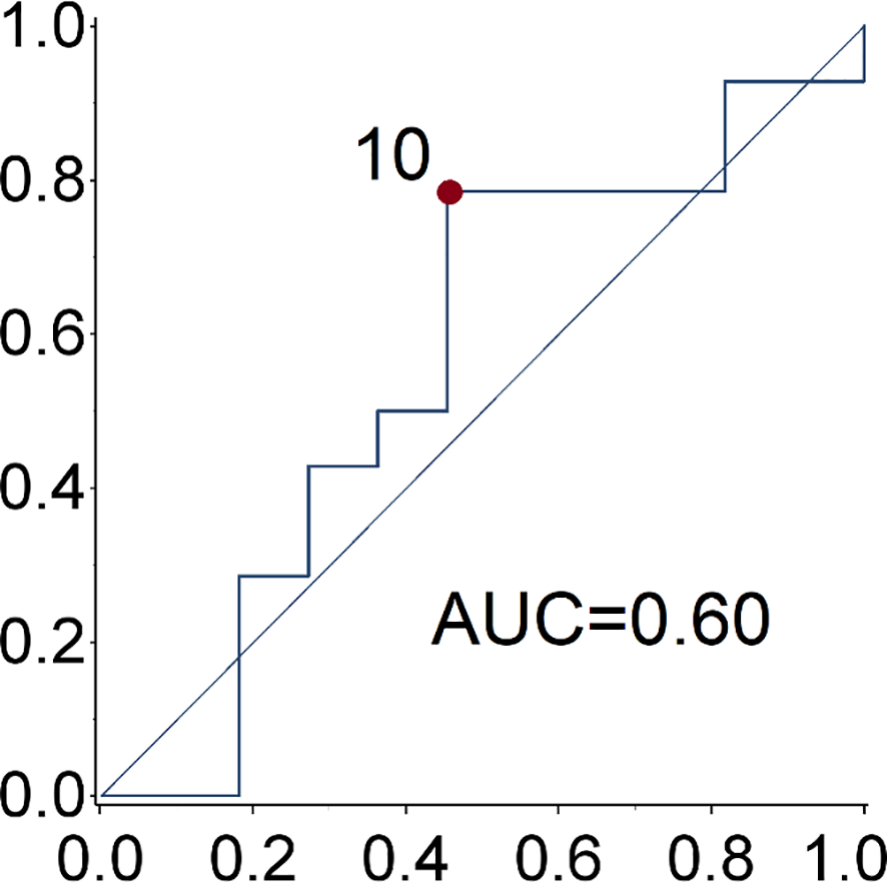
ROC curve of Ki67 for the prediction of disease progression. The optimal ki-67 cut-point maximizing the Youden index for the prediction of disease progression at 28.6 months (median progression free survival) is 10%.

Patient and tumor characteristics, the type of SSA (duration of treatment) and toxicities were described with conventional descriptive statistical analysis. Due to the explorative nature of this study, no sample size was determined. Any associations between clinical-pathological features and clinical benefit (partial response and stable disease) to the treatment was tested using the exacted Fisher test. The cut-off date for the analysis was June 2020. Receiver operating characteristic (ROC) curve analysis was used to identify the optimal cut-off value of Ki-67 for the prediction of disease progression (**Figure 1**). The PFS and OS curves were calculated with the Kaplan-Meier method and compared with the log-rank test. The prognostic value of age, gender, smoking exposure, histological subtype, Ki-67 category, surgery of primary tumor, functional tumor status, metastatic site, time between diagnosis and treatment start and type of SSA were evaluated in relation to PFS. The Cox regression model was used to estimate the hazard ratios (HR) and relevant 95% confidence intervals (CIs). Two-sided p-values are reported and a p-value of 0.05 was considered statistically significant. The statistical analyses were performed using the SAS software version9.4 (SAS Institute Inc. Cary NC, USA).

## Results

Data of thirty-one consecutive patients, meeting the eligibility criteria, were analyzed. The clinical and pathological and characteristics and type of SSAs administered are summarized in **Table 1**.

**Table 1:** Patient Characteristics.

PATIENTS CHARACTERISTICS	n
Patient number	31
Median age at SSA initiation	62 (range 20-82)
Gender	
Female	18 (58%)
Male	13 (42%)
Tobacco exposure	
Yes	1 (3%)
Previous	8 (25%)
Never	11 (32%)
ECOG PS	
0	30 (94%)
1	1 (6%)
TUMOR CHARACTERISTICS	
Histological subtype	
Typical carcinoid	14 (45%)
Atypical carcinoid	17 (55%)
Ki-67	
≤ 2%	5 (20%)
3-20%	19 (76%)
>20%	1 (4%)
Missing	6
Mitotic count	
< 2/2 mm2	8 (57%)
2-10/2 mm2	9 (53%)
Missing	14
Necrosis	
Negative	22 (96%)
Positive	1 (4%)
Missing	8
TTF1	
Negative	9 (29%)
Positive	11 (35%)
Primary tumor site	
Right	18 (58%)
Left	10 (32%)
Bilateral	3 (10%)
Functional status	
Carcinoid syndrome	6 (19%)
Non functioning tumors	25 (81%)
Metastases	
Synchronous	18 (58%)
Metachronous	13 (42%)
Site of metastases	
Liver	8 (26%)
Liver and other sites	11 (35%)
Bone	12 (39%)
Extra-regional nodes	6 (19%)
Lung	6 (19%)
Peritoneal	3 (10%)
Brain	1 (3%)
Others	6 (19%)
Number of metastases sites	
1 organ	16 (52%)
> 1 organ	15 (48%)
Somatostatin receptor positivity at nuclear medicine imaging	
Ga68-PET/CT	13 (42%)
Octreoscan	3 (10%)
TREATMENT CHARACTERISTICS	n (%)
Primary tumor resected	
Yes	18 (58%)
No	13 (42%)
Somatostatin analogs	
Octreotide LAR	24 (77%)
Lanreotide depot	7 (23%)

CI, confidence interval; LAR, long acting release; OS, overall survival; PFS, progression free survival; RECIST, response evaluation criteria in solid tumors; SSA, somatostatin analogue; LAR, long-acting release.

Thirteen (41.9%) patients were male, median age at SSA initiation was 62 years (range 20-82 years). The majority of patients (93.5%) had an ECOG PS 0.

Fourteen patients (45.2%) had a typical carcinoid, 17 (54.8%) an atypical carcinoid. Six patients had a carcinoid syndrome. One tumor, classified as atypical carcinoid, had Ki-67≥20%. This latter tumor was classified as an atypical carcinoid due to the well-differentiated phenotype. Necrosis was described in only one patient (3.2%). Radiological staging at diagnosis was performed with thoracic and abdominal CT in 17 (55%) patients. PET Gallium or Octreoscan were performed in 16 (51.6%) patients. All disease lesions displayed great uptake of the radiotracers.

Metastases were synchronous in 18 (58.1%) patients and metachronous in 13 (41.9%). Eighteen (58.1%) patients underwent surgery of the primary tumor which consisted in lobectomy in 3 patients (9.7%), lobectomy plus lymphadenectomy in 11 patients (35.5%) and atypical resection in 4 others. Liver was the most frequent metastatic site (61.3%). The other metastatic sites were bone (38.7%), contralateral lung (19.4%), extra-regional nodes (19.4%) and peritoneum (9.7%).

### Treatment Administered

Median time from diagnosis of primary neuroendocrine tumor to SSA therapy start was 13 months (range: 3-82). Twenty-seven (87.1%) patients received octreotide LAR, 26 at the standard dose of 30mg/4 weeks (one of them subsequently switched to a dose of 30mg/3 weeks), and one at standard dose of 20mg/4 weeks. Seven (22.6%) patients received lanreotide depot, six at standard dose of 120mg/28 days and one at 60mg/28 days. Three of them received both Octreotide and Lanreotide, in succession. The median treatment duration was 30 months (range: 3-82). Three patients (9.7%) developed mild diarrhea, no other adverse events were noted.

After a median follow up of 44 months (range 11-103 months), 20 patients (64.5%) underwent disease progression and 17 (54.8%) stopped the SSA therapy. Second line therapies were capecitabine plus temozolomide (CAPTEM regimen) in 7 patients, everolimus in 4 patients and peptide receptor radio-nuclide therapy (PRRT) in 4 patients. The remaining two patients received best supportive care. At the date of the last follow-up examination, 11 (35.5%) patients were free from progression and still under SSAs.

### Tumor Response, Progression-Free and Overall Survival

**Figure 2:**
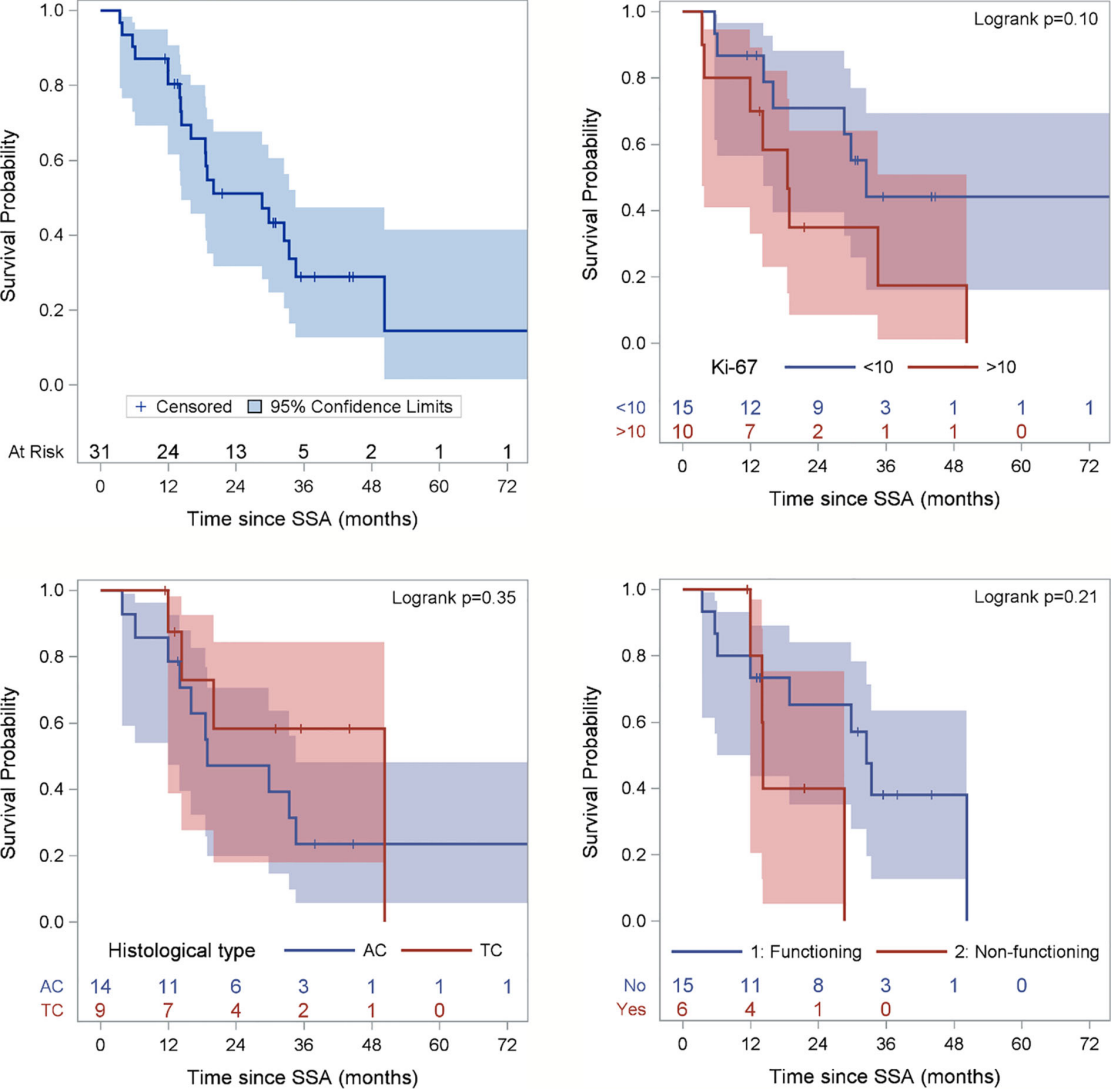
Progression-free Survival (PFS) of the entire population and according to Ki67 category, histological subtype and functioning status. Ki67 is missing for 6 patients and functioning status for 10 patients. Figure for histological type excludes 2 patients with NEN, 2 carcinoid tumors not otherwise specifies (NOS) and 5 well-differentiated NETS.

**Figure 3:**
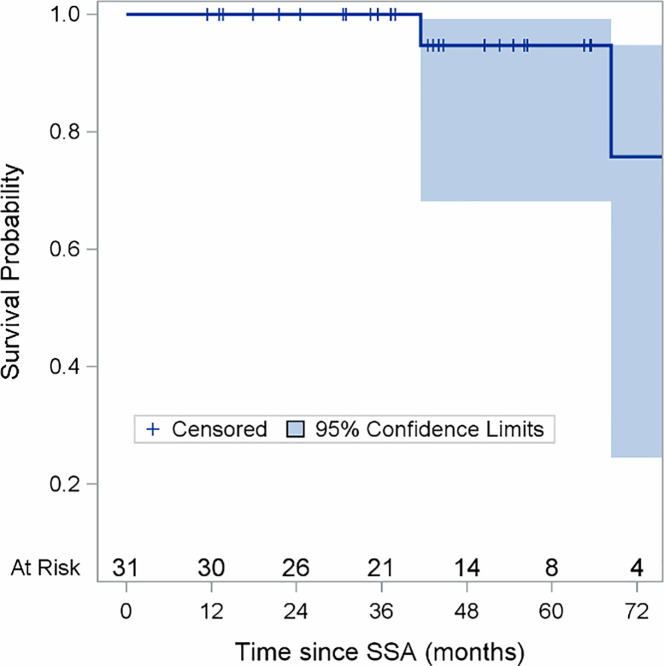
Overall Survival (OS) in the Entire Population.

Two patients (6.5%) obtained a partial response (PR), 24 (77.4%) stable disease (SD), whereas five patients (16.1%) had disease progression. After a median follow up of 44 months (range 11-103), the median PFS was 28.6 months (95% confidence interval (CI): 1.5-41.8 months) (**Figure 2**). At the last follow-up examination only two patients died so the median OS was not reached (**Figure 3**). All patients with carcinoids syndrome obtained an efficacious syndrome control (flushing and diarrhea), with complete symptom response in all of them.

A not significant more prolonged PFS was observed in patients with Ki-67 ≤ 10%, typical carcinoid, non-functioning disease, no liver metastases as opposed to their respective counterparts (**Table 2** and **Figure 2**).

**Table 2:** Studies data comparison.

Factor	Median PFS (months)	Hazard Ratio (95% CI)	P
Age<60 yr≥60 yr	27.721.4	0.71 (0.25-1.99)	0.51
GenderMaleFemale	23.324.5	1.07 (0.43-2.69)	0.62
Smoking exposureYesNo	22.528.5	1.45 (0.40-5.25)	0.57
Ki67≤10%>10%	27.619.9	0.42 (0.15-1.18)	0.23
Histologytypicalatypical	30.619.7	0.69 (0.27-1.72)	0.16
StatusFunctioningNon-functioning	17.027.3	2.57 (0.70-9.47)	0.17
Primary tumor surgeryYesNot	22.924.1	1.07 (0.43-2.65)	0.88
Time interval diagnosis -treatment start<1 yr≥1 yr	21.924.6	1.01 (0.43-2.65)	0.89
Liver metastasisYesNo	21.021.6	1.63 (0.35-7.63)	0.54
Metastasis sites1>1	31.917.4	2.88 (1.13-7.37)	0.19
Somatostatin analogsOctreotide LARLanreotide LAR	27.421.8	0.96 (0.27-3.50)	0.95

CI, confidence interval; LAR, long acting release; OS, overall survival; PFS, progression free survival; RECIST, response evaluation criteria in solid tumors; SSA, somatostatin analogue.

None of following parameters were associated with PFS: age (<60 vs ≥60 years), gender, smoking exposure, primary tumor surgery, site of metastasis, time interval between diagnosis and treatment start (<1 vs ≥1 year), SSA administered (octreotide vs lanreotide) (**Table 2**).

## Discussion

Somatostatin analogs are standard treatment in the management of well-differentiated neuroendocrine tumors of the gastro-entero-pancreatic tract, both functioning and non-functioning. On the basis of the results of the two prospective randomized studies (29, 30), their administration as first line approach is recommended to patients whose tumor expresses ki-67 in less than or equal to 10% of cancer cells (2). No prospective studies of first-line SSA have been conducted in patients with lung NET.

In this retrospective series the 2 SSAs: octreotide and lanreotide, were shown to be active as first line therapy in patients with advanced/metastatic lung NET with a proportion of partial responses and stable diseases not different from that obtained in the GEP NET setting. The good overall survival should be interpreted considering the short follow-up and the few events observed, that limit the generalization of this result. The 28-month PFS is promising, considering that lung carcinoids, in particular atypical ones, generally have a worse prognosis than GEP NET patients (2). The PFS of the present case series is superior to that of a published retrospective study of lung carcinoids submitted to front line SSAs (22). The difference can be attributed to the patient selection and in particular the higher proportion of typical carcinoids in our series, as compared to that of Bongiovanni A et al. (22).

As expected, atypical carcinoid was associated with a lower PFS than typical carcinoid, although not statistically significant. However, the 19 month PFS of patients with atypical carcinoid is a better outcome compared with historical reports (6). As mentioned in the introduction, the efficacy of SSAs was demonstrated in GEP NETs with Ki67 ≤10%. The observed median PFS of 27 months in lung carcinoid patients with Ki67<10% in the present paper is suggestive of efficacy of these drugs also in this subset. This series however included 9 patients whose lung carcinoid had a ki67 ranging between 11 and 20% and 1 patient with ki67 >20%. The median PFS of 19.9 months in patients with ki67 >10% is noteworthy and suggests that also selected patients with ki67 >10% could benefit from SSAs administered as frontline therapy. This observation needs confirmation in future prospective studies.

In our series the PFS was negatively influenced by the functional status (although without attaining the statistical significance) confirming a previous observation (22). However, it did not change dividing patients according to the tumor burden and the presence of liver metastases, suggesting that these parameters could not be considered a deterrent in the prescription of SSAs as first-line therapy.

## Conclusion

In summary, the long PFS obtained in this patient series with progressive, metastatic pulmonary NET, uniformly submitted to first line SSAs suggest that these drugs could be potentially efficacious in this patient setting, confirming previous observations (21, 22). These findings are relevant in patients with Ki67 ≤10% and typical carcinoids, but are encouraging also patients with greater Ki67 expression and atypical hystotype. Although no definitive conclusions can be drawn from our series of patients and that all our outcomes should be interpreted with caution, the results of our analysis are encouraging and should be considered as hypothesis-generating for further prospective studies.

## Data Availability Statement

The raw data supporting the conclusions of this article will be made available by the authors, without undue reservation.

## Ethics Statement

The studies involving human participants were reviewed and approved by ethics committee of European Institute of Oncology. Written informed consent for participation was not required for this study in accordance with the national legislation and the institutional requirements.

## Author Contributions

EL, ABe, NF, and FS contributed to conception and design of the study. EL organized the database. PM and AA performed the statistical analysis. AA, EL, and ABe wrote the first draft of the manuscript. All authors contributed to the article and approved the submitted version.

## Funding

This study was in part supported by The Italian Ministry of Health with Ricerca Corrente and 5 × 1000.

## Conflict of Interest

The authors declare that the research was conducted in the absence of any commercial or financial relationships that could be construed as a potential conflict of interest.

## References

[1] Korse CM Taal BG van Velthuysen MLF Visser O. Incidence and Survival of Neuroendocrine Tumours in the Netherlands According to Histological Grade: Experience of Two Decades of Cancer Registry. Eur J Cancer (2013) 49:1975–83. 10.1016/j.ejca.2012.12.022 23352435

[2] Baudin E Caplin M Garcia-Carbonero R Fazio N Ferolla P Filosso PL. Lung and Thymic Carcinoids: ESMO Clinical Practice Guidelines for Diagnosis, Treatment and Follow-Up. Ann Oncol (2021) 32(4):439–51. 10.1016/j.annonc.2021.01.003 33482246

[3] Ferguson MK Landreneau RJ Hazelrigg SR Altorki NK Naunheim KS Zwischenberger JB. Long-Term Outcome After Resection for Bronchial Carcinoid Tumors. Eur J Cardiothorac Surg (2000) 18:156. 10.1016/S1010-7940(00)00493-0 10925223

[4] Filosso PL Oliaro A Ruffini E Bora G Lyberis P Asioli S. Outcome and Prognostic Factors in Bronchial Carcinoids: A Single-Center Experience. J Thorac Oncol (2013) 8:1282. 10.1097/JTO.0b013e31829f097a 24457239

[5] Thomas CF Jr Tazelaar HD Jett JR. Typical and Atypical Pulmonary Carcinoids : Outcome in Patients Presenting With Regional Lymph Node Involvement. Chest (2001) 119:1143. 10.1378/chest.119.4.1143 11296182

[6] Lou F Sarkaria I Pietanza C Travis W Roh MS Sica G. Recurrence of Pulmonary Carcinoid Tumors After Resection: Implications for Postoperative Surveillance. Ann Thorac Surg (2013) 96:1156. 10.1016/j.athoracsur.2013.05.047 23915584

[7] Fiala P Petrásková K Cernohorský S Kinkor Z Krepela E Zatloukal P. Bronchial Carcinoid Tumors: Long-Term Outcome After Surgery. Neoplasma (2003) 50:60. 12687280

[8] Filosso PL Guerrera F Evangelista A Welter S Thomas P Casado PM. Prognostic Model of Survival for Typical Bronchial Carcinoid Tumours: Analysis of 1109 Patients on Behalf of the European Association of Thoracic Surgeons (Ests) Neuroendocrine Tumours Working Group. Eur J Cardiothorac Surg (2015) 48:441. 10.1093/ejcts/ezu495 25564217

[9] Rindi G Klimstra DS Abedi-Ardekani B Asa SL Bosman FT Brambilla E. A Common Classification Framework for Neuroendocrine Neoplasms: An International Agency for Research on Cancer (IARC) and WorldHealth Organization (WHO) Expert Consensus Proposal. Modern Pathol (2018) 31:1770–86. 10.1038/s41379-018-0110-y PMC6265262 30140036

[10] Skuladottir H Hirsch FR Hansen HH Olsen JH. Pulmonary Neuroendocrine Tumors: Incidence and Prognosis of Histological Subtypes. A Population Based Study Denmark Lung Cancer (2002) 37:127. 10.1016/S0169-5002(02)00080-6 12140134

[11] Cardillo G Sera F Di Martino M Graziano P Giunti R Carbone L. Bronchial Carcinoid Tumors: Nodal Status and Long-Term Survival After Resection. Ann Thorac Surg (2004) 77:1781. 10.1016/j.athoracsur.2003.10.089 15111186

[12] Soga J Yakuwa Y. Bronchopulmonary Carcinoids: An Analysis of 1,875 Reported Cases With Special Reference to a Comparison Between Typical Carcinoids and Atypical Varieties. Ann Thorac Cardiovasc Surg (1999) 5:211. 10508944

[13] Caplin ME Baudin E Ferolla P Filosso P Garcia-Yuste M Lim E. ; ENETS Consensus Conference Participants. Pulmonary Neuroendocrine (Carcinoid) Tumors: European Neuroendocrine Tumor Society Expert Consensus and Recommendations for Best Practice for Typical and Atypical Pulmonary Carcinoids. Ann Oncol (2015) 26(8):1604–20. 10.1093/annonc/mdv041 25646366

[14] National Comprehensive Cancer Network. Neuroendocrine and Adrenal Tumors (2020). Available at: https://www.nccn.org/professionals/physician_gls/pdf/neuroendocrine.pdfbone.pdf.

[15] Harpole DH Jr Feldman JM Buchanan S Young WG Wolfe WG. Bronchial Carcinoid Tumors: A Retrospective Analysis of 126 Patients. Ann Thorac Surg (1992) 54:50. 10.1016/0003-4975(92)91139-Z 1610254

[16] Beasley MB Thunnissen FB Brambilla E Hasleton P Steele R Hammar SP. Pulmonary Atypical Carcinoid: Predictors of Survival in 106 Cases. Hum Pathol (2000) 31:1255. 10.1053/hupa.2000.19294 11070119

[17] Kaplan B Stevens CW Allen P Liao Z Komaki R. Outcomes and Patterns of Failure in Bronchial Carcinoid Tumors. Int J Radiat Oncol Biol Phys (2003) 55:125. 10.1016/S0360-3016(02)03796-3 12504044

[18] Gould PM Bonner JA Sawyer TE Deschamps C Lange CM Li H. Bronchial Carcinoid Tumors: Importance of Prognostic Factors That Influence Patterns of Recurrence and Overall Survival. Radiology (1998) 208:181. 10.1148/radiology.208.1.9646811 9646811

[19] Fink G Krelbaum T Yellin A Bendayan D Saute M Glazer M. Pulmonary Carcinoid: Presentation, Diagnosis, and Outcome in 142 Cases in Israel and Review of 640 Cases From the Literature. Chest (2001) 119:1647. 10.1378/chest.119.6.1647 11399686

[20] Reidy DL Kulke MH Wolin EM Singh S Ferone D Mirakhur B. Safety and Efficacy of Lanreotide Depot/Autogel (LAN) in Patients With Lung NETs: The Randomized, Double-Blind, Placebo (PBO)-Controlled Phase III SPINET Study. J Clin Oncol 34(15_suppl). 10.1200/JCO.2016.34.15_suppl.TPS8580

[21] Sullivan I Le Teuff G Guigay J Caramella C Berdelou A Leboulleux S. Antitumour Activity of Somatostatin Analogues in Sporadic, Progressive, Metastatic Pulmonary Carcinoids. Eur J Cancer (2017) 75:259. 10.1016/j.ejca.2016.11.034 28242503

[22] Bongiovanni A Recine F Riva N. Autcome Analysis of First-Line Somatostatin Analog Treatment in Metastatic Pulmonary Neuroendocrine Tumors and Prognostic Significance of FDG-PET/TC. Clin Lung Cancer 10(4):415–20. 10.1016/j.cllc.2016.11.004 27956089

[23] De Dosso S Bajetta E Procopio G Cortinovis D Buzzoni R Catena L. Pulmonary Carcinoid Tumours: Indolent But Not Benign. Oncology (2007) 73(3-4):162–8. 10.1159/000127382 18418008

[24] Srirajaskanthan L Watkins J Marelli L Khan K Caplin ME. Expression of Somatostatin and Dopamin2 Receptors in Neuroendocrine Tumors and the Potential Role for New Biotherapies. Neuroendocrinology (2009) 89(3):308–14. 10.1159/000179899 19307732

[25] Laskaratos FM Walker M Naik K. Predictive Factors of Antiproliferative Activity of Octreotide LAR as First Line Therapy for Advanced Neuroendocrine Tumors. Br J Cancer (2016) 115(11):1321–7. 10.1038/bjc.2016.349 PMC5129835 27811856

[26] Ozaslan E Karaca A Koca S. Comparison of Survival With Somatostatin Analog and Chemotherapy and Prognostic Factors for Treatment in 165 Advanced Neuroendocrine Tumors Patients With Ki-67 20% or Less. Anticancer Drugs (2017) 28(2):222–9. 10.1097/CAD.0000000000000445 27768606

[27] Dasari A Bergsland E Benson A. Treatment Patterns and Clinical Outcomes in Advantage Lung Neuroendocrine Tumors in Real World Settings: A Multicentre Retrospective Chart Review Study. Oncologist (2019) 24(8):1066–75. 10.1634/theoncologist.2018-0520 PMC6693720 30610008

[28] Forde PM Hooker CM. Advanced Pulmonary Carcinoid (APC): 20-Year Experience At Johns Hopkins (Jh). J Clin Oncol (2013) 31:15 SUP. 10.1200/jco.2013.31.15_suppl.7598

[29] Rinke A Müller HH Schade-Brittinger C Klose KJ Barth P Wied M. Placebo-Controlled, Doubleblind, Prospective, Randomized Study on the Effect of Octreotide LAR in the Control of Tumor Growth in Patients With Metastatic Neuroendocrine Midgut Tumors: A Report From the PROMID Study Group. J Clin Oncol (2009) 27:4656. 10.1200/JCO.2009.22.8510 19704057

[30] Caplin ME Pavel M Ruszniewski P. Lanreotide in Metastatic Enteropancreatic Neuroendocrine Tumors. N Engl J Med (2014) 371:1556. 10.1056/NEJMoa1316158 25317881

